# Efficient and Reliable Geocoding of German Twitter Data to Enable Spatial Data Linkage to Official Statistics and Other Data Sources

**DOI:** 10.3389/fsoc.2022.910111

**Published:** 2022-06-09

**Authors:** H. Long Nguyen, Dorian Tsolak, Anna Karmann, Stefan Knauff, Simon Kühne

**Affiliations:** Faculty of Sociology, Bielefeld University, Bielefeld, Germany

**Keywords:** Twitter, geocoding, spatial linkage, official statistics, regional analysis

## Abstract

More and more, social scientists are using (big) digital behavioral data for their research. In this context, the social network and microblogging platform Twitter is one of the most widely used data sources. In particular, geospatial analyses of Twitter data are proving to be fruitful for examining regional differences in user behavior and attitudes. However, ready-to-use spatial information in the form of GPS coordinates is only available for a tiny fraction of Twitter data, limiting research potential and making it difficult to link with data from other sources (e.g., official statistics and survey data) for regional analyses. We address this problem by using the free text locations provided by Twitter users in their profiles to determine the corresponding real-world locations. Since users can enter any text as a profile location, automated identification of geographic locations based on this information is highly complicated. With our method, we are able to assign over a quarter of the more than 866 million German tweets collected to real locations in Germany. This represents a vast improvement over the 0.18% of tweets in our corpus to which Twitter assigns geographic coordinates. Based on the geocoding results, we are not only able to determine a corresponding place for users with valid profile locations, but also the administrative level to which the place belongs. Enriching Twitter data with this information ensures that they can be directly linked to external data sources at different levels of aggregation. We show possible use cases for the fine-grained spatial data generated by our method and how it can be used to answer previously inaccessible research questions in the social sciences. We also provide a companion R package, nutscoder, to facilitate reuse of the geocoding method in this paper.

## 1. Introduction

Computational approaches that incorporate large volumes of online data and related methods into substantive research have become increasingly popular in the social sciences. There is now a rapidly growing literature which studies the use of digital trace data or big data for their use in social science projects (Jungherr, 2018; Stier et al., 2019; Choi, 2020). Within this literature, researchers have pointed to a number of issues that afflict many novel data types and online sources (Amaya et al., 2020; Sen et al., 2021).

Twitter is one of the most common sources for digital trace data and has been used extensively by social scientists as well as other researchers. Twitter is a microblogging platform launched in 2006 that allows users to publicly share short texts, images, or videos and to connect to and follow other users in professional or private networks. For researchers, Twitter is of particular interest, as its data is comparatively easy to access and collect (McCormick et al., 2015). Using Twitter data, researchers can study both the content of communication on Twitter—for example, by applying natural language processing techniques to large text corpora (e.g., Lwin et al., 2020)—as well as meta-information about the platform, usually to analyze networks of users (e.g., Ahmed et al., 2020). Applications of Twitter data analysis have been published in fields including political science, sociology, communication science, and public health studies (for an overview of research with Twitter data, see Karami et al., 2020).

One promising use of Twitter (meta) data is the analysis of geospatial information that accompanies tweets or user profiles (see Rieder and Kühne, 2018). Similar to research using regional properties to study survey respondents' living conditions (e.g., in urban sociology), research using Twitter data can examine the spatial distribution of tweets, compare the content of tweets across regions, or link Twitter data with external data sources by way of regional identifiers to study a variety of phenomena. Recent studies in the social sciences have used Twitter geoinformation to study the COVID-19 pandemic (Ntompras et al., 2022), influenza trends (Gao et al., 2018), crime (Hipp et al., 2018), language dialects (Huang et al., 2016), conspiracy theories (Stephens, 2020), polling (Beauchamp, 2017), travel and mobility (Blanford et al., 2015; Zhang et al., 2017; Wang et al., 2018; Levy et al., 2020), health behavior and outcomes (Wiedener and Li, 2014; Nguyen et al., 2017; Martinez et al., 2018), anti-immigrant attitudes (Menshikova and van Tubergen, 2022), happiness (Mitchell et al., 2013), and human behavior in environmental disasters (Murthy and Gross, 2017).

However, despite the vast amount of data, ready-to-use geospatial information—in the form of geographic coordinates—is only available for a small fraction of tweets. The majority of users choose not to provide the social network with GPS^1^ access to their devices when sending tweets. Sloan and Morgan (2015) estimated the share of users who allow geotagging by Twitter to be 3.1%. At the tweet level, Sloan et al. (2013) estimated the share of geotagged tweets to be 0.85%. These results are supported by our analysis of over 866 million German tweets, in which the shares of tweets and users with Twitter geotags are 0.18 and 0.31% respectively. As a result, only a very small portion of Twitter data can be readily combined with external information about geographical areas, limiting the potential applications and increasing the threat of bias in estimates based on the data. For the latter, we already know from existing studies that in many countries, on average, Twitter users are more likely to be male, younger, more highly educated, wealthier, and to live in urban as opposed to rural areas (Blank, 2017; Yildiz et al., 2017; Beisch and Koch, 2021). Blank (2017) also points to systematic differences in online behaviors and attitudes that dramatically limit the potential for social science research when seeking to provide estimates for larger social groups (or even the general population). Further, Sloan and Morgan (2015) highlight additional biases in working with geotagged Twitter data by comparing users who allow geotagging of their tweets to those who do not: male and older users are more likely to share geotags and more likely to show a different set of languages in their tweets.

Clearly, adding missing but needed geographic information will increase the proportion of tweets or users that can be attributed to geographic regions, which will improve the usability of Twitter data for the study of regional context effects. In this paper, we propose a method to reliably and efficiently leverage the user-supplied free text in the location field of Twitter profiles to retrieve geographic locations as an alternative to the GPS geotags provided by Twitter. Since there are many more Twitter users who specify their profile locations than those who enable geotagging via GPS, this strategy can make a much larger portion of Twitter data usable for geospatial analysis, potentially decreasing the population bias in geotagged tweets for the analysis of regional relationships (Malik et al., 2015). Although profile locations are readily available along with tweet data, the challenge—due to the nature of the data as free text—is generally to identify as many real locations as possible while filtering out nonsensical or nonexistent locations (Hecht et al., 2011).

**Figure 1:**
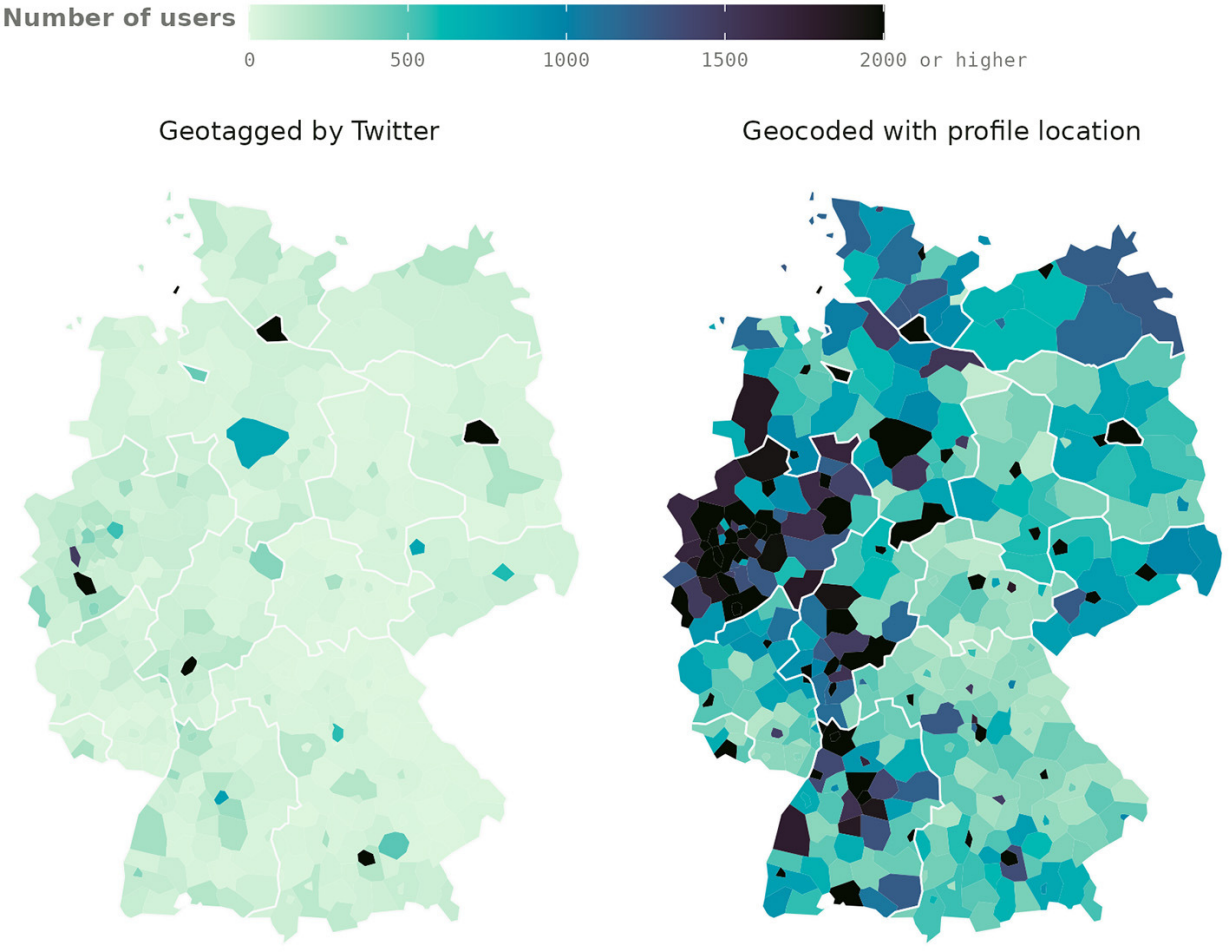
Number of Twitter users who tweeted (retweets and tweets from verified accounts not included) between October 15, 2018, and October 14, 2021, per NUTS-3 region in Germany according to Twitter geotags and our geocoding results using user profile locations.

In addition to identifying real-world places that correspond to Twitter profile locations, we match them to (e.g., administrative) regions at different levels of spatial aggregation. Enriching Twitter data with this information ensures that it can be linked directly to regional data from other sources, such as official statistics. Figure 1 shows the increase in the number of geolocated users achieved by our method, aggregated at the NUTS-3^2^ level. While we focus on the specific case of German tweets and German administrative regions throughout this paper, our approach can easily be applied to other countries as well.

Building on our process of geocoding Twitter profile locations, we also provide nutscoder—a free, open-source software package in the R programming language—to help researchers implement our method in their analyses. To evaluate the results of our geocoding method, we a) assess the accuracy of the geocoded locations based on four common token-based and distance-based evaluation metrics and also compare, b) the spatial distribution of our geolocated tweets against the distribution of tweets geotagged by Twitter with respect to the distribution of real-world population as well as, and c) the content of geolocated and non-geolocated tweets using a bag-of-word approach. Finally, we demonstrate the potential of our geocoded data for regional analyses in several use cases.

## 2. Geolocation of Twitter Data: Background and Related Work

Regional analyses using Twitter data require data to be mapped to real locations of the world. Locations of tweets and users can be derived based on a variety of sources within Twitter data. The sources commonly used to locate Twitter users and tweets can be divided into three categories: a) Twitter metadata, b) Twitter user networks, and c) content of tweets (Miura et al., 2017; Zheng et al., 2018).

### 2.1. Twitter Metadata

Metadata is the data that accompany a tweet when a user posts it. A tweet's metadata includes information about the tweet, such as timestamp and information about the user, such as their display name and profile location text as well as GPS geotag (if available). Among these, GPS geotags are the most obvious source of location information, as they come in the form of geographic coordinates (longitude and latitude) and represent precise locations on the Earth's surface without any further processing. Thanks to their ease of use, tweet geotags are utilized by many researchers to locate tweets and users in their analysis (Mitchell et al., 2013; Hawelka et al., 2014; Wiedener and Li, 2014; Blanford et al., 2015; Shelton et al., 2015; Huang et al., 2016; Murthy and Gross, 2017; Nguyen et al., 2017; Zhang et al., 2017; Hipp et al., 2018; Martinez et al., 2018; Wang et al., 2018; Levy et al., 2020). However, this information is available for not even 1% of all tweets (Sloan and Morgan, 2015). Consequently, studies using exclusively tweets that are geotagged by Twitter limit themselves to a tiny subsample of the available data. Furthermore, the potential for more more granular regional analysis is severely restricted due to the small number of tweets available per spatial unit of analysis.

Twitter metadata provides another source for geographic locations in the user profile location field. This information is available for about two thirds of all users (Alex et al., 2016)^3^, indicating the potential for much better coverage. Similar to tweet geotags, user profile locations are also intended to provide specific geographic information. Many studies to date have used location information derived from profile locations to supplement the information given by GPS geotags and provide a better sample size for analysis (Beauchamp, 2017; Stephens, 2020; Ntompras et al., 2022).

However, since user profile locations are simply free text fields for which Twitter has no constraints with regard to their correctness, many users misuse this field to state information that has nothing to do with their locations (Hecht et al., 2011). On the other hand, valid location names can take many forms due to, for example, abbreviation, capitalization, punctuation, and the order of the components of a place name. A method for geolocation based on user profile locations must therefore be able to recognize as many valid locations as possible among all available profile location text strings.

An obvious strategy for studies that use Twitter profile locations for geolocation is to employ pattern matching, for example, using regular expressions (regex), to assign profile location text to real-world location (e.g., Beauchamp, 2017). The challenge with this approach is twofold. First, the list of real location names must be large enough to cover all the regions in which the researcher is interested. This means not only having all the desired target regions, but also as many places as possible within those regions. For example, a researcher who wants to locate users in the state of Bavaria and only has the state's name in their reference list of places to match to Twitter profile locations will miss users who do not explicitly have “Bavaria” in their profile, but only the names of cities within the state such as “Munich” or “Nuremberg.” Second, creating regex patterns that can reliably accommodate all possible variations in the spelling of place names is an almost impossible task. Thus, studies using *ad hoc* regex searches on user profile locations for real-world location detection are at risk of missing a significant proportion of valid location strings.

Alex et al. (2016) demonstrates a more complex approach for geolocation based on user profile locations. In this method, the Edinburgh Geoparser (Grover et al., 2010), which uses lexicon-based and rule-based named entity recognition and was originally developed to find real-world locations in regular running English text, is adapted to geolocate Twitter profile location strings and shows promising results (Alex et al., 2016). Also using specialized software—in this case, Yahoo's PlaceFinder API—to extract real-world locations from profile location text, Dredze et al. (2013) constructed a pipeline that is fast enough to return users' geographic locations in real time, proving useful for disease surveillance systems. Other applications of dedicated geolocation services and databases in the literature include the use of the Google Geocoding API and GeoNames^4^ (Stephens, 2020; Ntompras et al., 2022). However, all these services are subjected to usage fees and/or restrictions regarding the size of the target name list as well as the speed of queries.

### 2.2. Twitter User Networks

GPS geotags and user profile locations cover the scope of Twitter data intended for the purpose of geolocation. In cases where these two pieces of information are not available, researchers must rely on other parts of Twitter data that do not explicitly refer to geographic locations but may still help to predict this information. The first of the two major approaches of this kind involves exploiting user networks—formed by interactions between Twitter users, such as following or mentioning one another—as a basis for inferring user locations. Simply put, network-based geolocation methods use available geographic information about users in a network and their relationships to predict geographic information for users for whom geographic information is not available in their metadata. This strategy relies on the assumption that users residing within the same area are more likely to communicate frequently (Ajao et al., 2015). While this is generally true (McGee et al., 2011, 2013; Jurgens, 2013), the likelihood of interactions between users also depends on a multitude of other factors, for example, users' popularity and topics of interest (Chandra et al., 2011; Li et al., 2012). A great number of methods have been developed to draw predictions about users' locations from their interaction networks (and the geographic information available from the aforementioned metadata for users in their networks), which typically involve probabilistic and machine learning models that incorporate the available spatial and network data (Backstrom et al., 2010; Davis Jr. et al., 2011; Jurgens, 2013; Rout et al., 2013; Cheng et al., 2014; Compton et al., 2014; Kong et al., 2014; Ghoorchian and Girdzijauskas, 2018). However, such methods cannot easily be scaled to real-world applications and their performance varies greatly depending on the geographic information available to be used as ground truth (Jurgens et al., 2015).

### 2.3. Content of Tweets

The final frequently used source of geographic information about Twitter data is the content of a tweet itself. This approach applies natural language processing methods on the text of a tweet to predict user location by leveraging words indicative of locality, for example, by being more commonly used in certain regions. Due to the unstructured nature of the data and the general complexity of the problem, geolocation methods using tweet content employ a wide range of techniques, ranging from maximum likelihood approaches to machine learning/deep learning models, both supervised and unsupervised (Cheng et al., 2010; Chandra et al., 2011; Wing and Baldridge, 2011; Roller et al., 2012; Han et al., 2013, 2014; Graham et al., 2014; Onan, 2017; Hoang and Mothe, 2018). Obviously, geolocation methods can also combine tweet content, including photos (Matsuo et al., 2017), with network data and metadata to achieve better results (Ren et al., 2012; Elmongui et al., 2015; Miura et al., 2017; Bakerman et al., 2018; Ribeiro and Pappa, 2018; Tian et al., 2020).

Compared to geolocation methods based on Twitter metadata, methods based on user networks and tweet content are more complicated because these data are not exclusively related to geographic locations, and thus geographic information in these data is sparse. Consequently, the results of network-based and content-based geolocation methods are highly uncertain in nature and generally less accurate. These methods therefore also require much more effort to validate and evaluate. Since the goal of our paper is to develop a method to geolocate data in a very large corpus of tweets in a reliable and efficient manner, Twitter metadata is the more suitable source of geographic information on which to base our method.

## 3. Data

### 3.1. Data Collection

Data collection in from the official Twitter API started on October 5, 2018, and is still ongoing. In our queries to the Twitter API^5^, we request real-time tweets that are tagged as German by Twitter's language detection and contain one of the 100 most common words—excluding punctuations and separators—in the German language^6^. The Twitter API requests return on average about 15 tweets per second (with some day-night cycle fluctuation), which amounts to 35–40 million tweets per month. While the Twitter API has a rate limit of 1% of all Twitter traffic globally, we believe this does not affect our data collection. Tromble et al. (2017) estimated the global rate to be 6,000 tweets per second in 2016, and based on Twitter's growth from 2016 to the present, we expect the amount of data that we collect to be well below the possible rate limit of about 60 tweets per second (1% of 6,000).

### 3.2. Dataset

Until March 2022, we have collected over 1.1 billion tweets (including retweets^7^). For the analysis in this paper, we use a subset over the 3-year period from October 15, 2018, to October 14, 2021. This subset does not include retweets. It also does not include tweets from so-called verified accounts, as these are mostly run by representatives of media and other organizations whose tweets tend to be neutral reporting of news and thus less interesting for our substantive applications in researching public attitudes and behaviors on the platform. With this restriction, our analysis sample consists of over 866 million tweets from 16.6 million users. Alongside the text of each tweet, the Twitter API provides additional information about the tweet, including a unique ID, the time of posting, the location of the device as a geographic coordinates, if available, and whether it was a retweet, as well as information about the user who posted the tweet, including a unique user ID, their username, follower count, profile description, and profile location, if available.

In order to link the data in the tweets with external data about geographical regions for use in regional analysis, we need an attribute that identifies the regions to which a tweet or its user can be assigned. When users give permission, Twitter collects their location in the form of geographic latitude and longitude. Researchers can easily pinpoint the location to which the specific latitude and longitude refer and choose the appropriate level of spatial and/or political aggregation—municipality, county, district, or state—to link the Twitter data with data from other sources.

In our dataset, however, only about 1.53 million or 0.18% of the tweets collected were tagged with geographic coordinates by Twitter. These geotagged tweets came from 51,180 Twitter users, or 0.31% of all the users in our analysis sample. This represents an even smaller amount of geographic information collected and shared by Twitter than what was reported in Sloan and Morgan (2015). This difference could be attributed to the fact that we only analyze German-language tweets, since users in Germany tend to be less willing than users in other countries to share geolocation information with their tweets (Scheffler, 2014).

If we use only those tweets in our dataset that were already geotagged by Twitter, we cannot perform meaningful regional analysis at the level of (and below) major cities (*kreisfreie Städte*) or counties (*Landkreise* or *Kreise*) in Germany. For many regions, the number of users who have at least one tweet with GPS coordinates falls in the low double-digit range or even below, with the lowest number being six (Table 1).

**Table 1:** NUTS-3 regions with the fewest users based on Twitter geotags.

**NUTS-3**	**Name**	**Users**
DEB3G	Kusel	6
DEG0D	Sömmerda	9
DE255	Schwabach	9
DE272	Kaufbeuren	10
DE22C	Dingolfing-Landau	11
DE247	Coburg	11
DE926	Holzminden	11
DE267	Haßberge	11
DEG0N	Eisenach, Stadt	11
DE234	Amberg-Sulzbach	12
DE23A	Tirschenreuth	12
DEB37	Pirmasens, kreisfreie Stadt	12
DEG06	Eichsfeld	12
DEG0A	Kyffhäuserkreis	12

An alternative source of geographic information in Twitter data that is also easily accessible and can be exploited to increase the number of geolocated tweets is the profile's location field, in which Twitter users can enter an arbitrary text that will be displayed publicly. Assuming that the text in the profile location corresponds to a user's actual location, this information has the potential to make a much larger portion of Twitter data usable for regional analysis. In contrast to the low percentages of tweets and users with Twitter geotags, 569 million (65.66%) of our 866 million tweets (excluding retweets and tweets from verified accounts) collected during the 3-year period were posted by users who had entered something in the location field of their profiles. These users (9.2 million) make up 59.15% of the total number of users in our analysis sample (16.6 million users).

However, it has to be noted that not every Twitter user who uses the profile location field uses it for its designated purpose, as users can enter any text string 30 characters or shorter in this field. For example, many write indecipherable sequences of letters and emojis. Many others misuse this space to make their age and/or gender pronouns known. Other examples of non-location strings that users give as their location are “mind your own business,” “dying of hunger,” and “goat cheese radish tartine^8^.”

**Figure 2:**
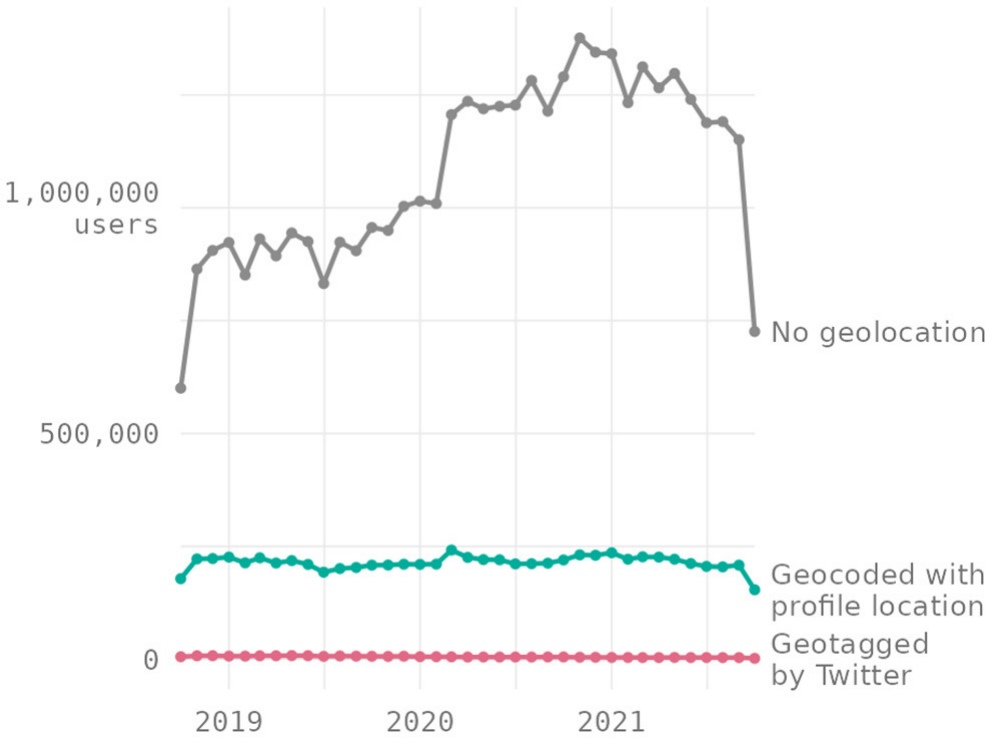
Number of monthy active Twitter users in our dataset.

Figure 2 shows the number of monthly active users over the 3-year period in our analysis dataset, grouped by the source of geospatial information about their tweets: from Twitter's geotags, geocoded based on their profile location, or none at all. For every month, we count as active users those who posted at least once tweet during the month^9^. Across each month, the number of active users in our German Twitter dataset who could be geocoded via their profile locations is much higher than the number of users whose tweets were geotagged by Twitter, while most users, however, could not be assigned a location in Germany. Note that users are grouped by whether they could be geolocated, so the number of Twitter users with geocoded profile locations is lower than the number of users with a location text in their profiles presented above. Also, while the number of users without geographic locations shows a general upward trend with a significant jump in early 2020, this trend is not observed for the number of users with geographic locations^10^.

## 4. Geocoding Twitter Profile Locations

### 4.1. Objectives

As mentioned earlier, metadata in the form of GPS coordinates needs virtually no processing, but is only available for a tiny fraction of all available tweets. Therefore, the purpose of our approach is to supplement this information with geographic information obtained from the profile location text, which is available for a large proportion of the data. Unlike geographic coordinates, locations as text strings need to be preprocessed in order to compile unambiguous geographic information, because a given place on Earth may be referred to in many ways. The process of extracting geographic information from text is called *geocoding*. Since geographic locations are unique and can often be identified as such, for example, in official statistics, free text locations in Twitter profiles need to be geocoded to enable a linkage of regional data with other data sources, which may then be leveraged for regional analysis.

A primary goal of our geocoding procedure is to discern—whenever possible—a corresponding spatial reference for a given location name in a Twitter user's profile. This means, on the one hand, that geocoding should allow for a variety of names that each location may be associated with. For example, we should be able to identify a Twitter user from the German city of Hamburg if they have a profile location that reads “Hamburg” or “HH” (its ISO code), or “Free and Hanseatic City of Hamburg” (its full official name). The language used for a place's name should also not influence where the place actually is: “Freie und Hansestadt Hamburg” (German), “Friee un Hansestadt Hamborg” (Low Saxon), “Hampuri” (Finnish), “Amburgo” (Italian), and “ハンブルク” (Japanese) should all be recognized as the same city. Furthermore, the geocoding results should not be dependent on the use of capitalization, punctuation, spaces, or the order of components in the location strings: “münchen,” “MÜNCHEN BY,” “München, Deutschland,” and “Germany / Bavaria / Munich” should all be assigned the same spatial reference. Likewise, geocoding should also be insensitive to additional non-text elements in the location string, such as emojis and other special Unicode characters. On the other hand, the geocoding rules must also be strict enough so as not to mistake non-locations that users enter in their profile, such as those listed in Section 3, for real locations.

A second important objective of our geocoding procedure is to make it easy to determine whether an observation can be included in aggregated statistics at a certain level of spatial aggregation. In contrast to Twitter's geographic tagging with the use of GPS, the name of a region can only reveal its shape as a polygon on the surface of the Earth, but not an exact point, since a region spans a larger area. For an exact point on the Earth's surface, the associated data can be aggregated to any higher or lower regional level that encompasses that point. For polygons, however, the lowest possible level of spatial aggregation is their own boundary. Knowing the lowest possible level of aggregation for each region as well as the encompassing regions at higher levels of aggregation is important to identify the appropriate spatial reference that can be used to link Twitter data with data from other (e.g., administrative) sources, as data about regions at a lower level can be aggregated to a higher level, but data about a region at a higher level cannot be easily disaggregated to regions at a lower level. For example, if a user's profile location says “Munich,” it is also non-problematic to use this observation as a part of the federal state of Bavaria, Germany in an analysis at the state level; however, the reverse is not true, since not every part of Bavaria is within the city of Munich, and a user with a profile location that says Bavaria cannot be part of an analysis of cities or other types of spatial units that are at a lower level of aggregation than federal states.

The sheer amount of data available (see Section 3) leads to an additional objective for our geocoding procedure: In order to make use of the geocoding results in our substantive research, we need to achieve the aforementioned goals for all of our collected tweets in a reasonable time. Additionally, as the data collection is ongoing, our geocoding tool chain should also be able to continuously process the new profile locations associated with the incoming tweets while avoiding repetitive geocoding of already processed locations to save time and computing resources, enabling us to establish a real-time pipeline for geocoding the collected Twitter data.

### 4.2. Implementation

Geocoding—the identification of geographic information based on the name of a place—is a common practice in spatial analysis that emerged and has continued to be refined over the last several decades (Goldberg et al., 2007). There are now a wide range of vendors and services available to facilitate the geocoding process, including free, open-source software solutions as well as enterprise-level products at global conglomerates like Google (Google Maps, 2022).

For our application, we opted for the open-source geocoder Nominatim, which allows users to search all of OpenStreetMap data (Nominatim, 2022b). OpenStreetMap is an initiative whose diverse contributors create and provide free geographic data about places all over the world (Map Foundation, 2021). By virtue of being free, open-source, and actively developed by a large community, both OpenStreetMap data and its search engine Nominatim offer themselves as a viable long-term solution for our purpose. Another advantage of Nominatim is the ability to geocode place names not only in English or the language of the country where a place is located, but also in many different other languages, especially for widely known place names.

Nominatim's search engine takes a text string as input and returns geographic information as well as other data from OpenStreetMap about the place in real life that corresponds to the input string. Thanks to sensible tokenization and normalization of OpenStreetMap place names as well as search input, Nominatim's text search engine can handle users' queries flexibly, also being tolerant of fuzzy matches and abbreviations (Hoffmann, 2021a,b; Nominatim, 2022d). Nominatim also provides a public instance at nominatim.openstreetmap.org, accompanied by an API that allows users to programmatically search for places in the OpenStreetMap database (Nominatim, 2022a).

It is important to note that Nominatim can return multiple places based on a given text string. This often occurs when there are multiple places with the same name, such as the US city of New York and the Munich hair salon named New York. In such cases, the places in the results are assigned a ranking based on Nominatim's internal search rank (e.g., a state has a higher search rank than a city, which has a higher rank than a suburb) or—when available—the Wikipedia importance ranking (Nominatim, 2022c). The latter is a function of the number of Wikipedia articles that are linked to a place's Wikipedia article (Nominatim, 2021). For our application, we limit the geocoding results to the first-ranked place that Nominatim returns for each location string.

By taking a list of all unique profile location strings that appear in our database, we reduce the number of cases for geocoding from 569 million tweets sent by users with a location in their profile to over 6 million location strings. After geocoding, the results can be joined back to user profiles via the location strings. However, despite the substantial reduction in the number of cases, the rate limit of 1 query per second of the public Nominatim server means that it would take us over 2 months to geocode the 6 million text strings that we have.

To overcome this problem, we host our own instances of Nominatim's database on our on-premise high-performance computing server (on which the relational database that contains all collected Twitter data is also hosted). More specifically, we deploy two Nominatim instances^11^: the first contains data for German places only and acts as a quick filter; the second covers the whole world and is used to perform the final geocoding step on the filtered profile location strings^12^. Not only does self-hosting free us from the query rate limit of the public Nominatim, it also enables complete access to Nominatim's database backend. The benefits of this are two-fold. First, we can exclude irrelevant places on the globe from the database, thus reducing the size of the database and making queries faster. Second, since this allows us to perform geocomputational operations such as spatial joins directly on objects in the database, we have flexible control over the geographic information that Nominatim queries return and are able to streamline it to our needs.

To preserve user privacy, we exclude the geocoding results in which the location text is matched with a place at the street address level, with the exception of train stations. This also greatly reduces the number of mishits, which are particularly prevalent for places at this level, as location strings containing common nouns are often matched with businesses such as shops and restaurants. For example, a user can specify their profile location as “Saturn” (presumably the planet), which is also the name of a chain of electronics stores in Germany and Luxembourg. Since there is no other place in Germany with a higher ranking that is also named Saturn, Nominatim will return the address of the Saturn store in Senden, Bavaria, which is the first-ranked result when searching for “Saturn.”

In addition to geocoding the profile locations and retrieving the geographic information about the place that corresponds to each location, we create a dataset that contains the official names and codes of administrative regions at different levels in Germany as well as the geographic geometries (also commonly known as “shapes”) of these regions. By performing spatial joins of the geocoded places' shapes on the shapes of the administrative regions, we can determine all administrative regions at different levels to which a geocoded place can be assigned, as well as the lowest administrative level at which analysis can be done with the geocoded data. More precisely, a Twitter profile location is matched to an administrative region if the place that corresponds to this location lies completely within the boundaries of that region. For example, in addition to being assigned to the city of Munich, a user whose profile location reads “Munich, Germany” is also matched with the state of Bavaria as well as any administrative region that completely encompasses Munich.

Since our analysis only deals with Twitter users in Germany, only the geometries of German regions are included in the target dataset for the spatial joins. This means that profile locations referring to actual places outside of Germany such as “Vienna, Austria” are excluded from the final results, as no administrative region in Germany covers Vienna on the map. Table 2 shows a sample of location strings and the NUTS codes of the regions that we could match with these strings using the described procedure.

**Table 2:** Random sample of geocoding results where the input is the Twitter profile location and the output is the corresponding administrative regions in Germany.

**Profile location**	**NUTS-1**	**NUTS-2**	**NUTS-3**
fRaNkFuRt	DE7	DE71	DE712
Aicha vorm Wald	DE2	DE22	DE228
Schwei	DE9	DE94	DE94G
Brochenzell	DE1	DE14	DE147
hh	DE6	DE60	DE600
nrw	DEA	–	–
Jena, Germany	DEG	DEG0	DEG03
Aub, Deutschland	DE2	DE26	DE26C
Germany-Mülheim an der Ruhr	DEA	DEA1	DEA16
Kuhbach im Schwarzwald	DE1	DE13	DE134

To facilitate automation of the geocoding process and make it reusable in other research, we create the R package nutscoder, which makes it straightforward to perform the described geocoding steps to generate corresponding administrative region codes from location names as free text. nutscoder also generalizes our geocoding practice so that it is applicable not only to Twitter profile locations, but to any text strings that refer to real-world locations. With the ability to customize the target dataset of administrative regions, the same procedure can also be used to geocode locations outside of Germany. Without access to our private server, however, nutscoder can only use the public Nominatim server (or an instance of the Nominatim database and API self-hosted by the package users). The package is publicly available and can be installed from github.com/long39ng/nutscoder.

### 4.3. Results

In total, we are able to match German administrative regions to over 74,000 of the unique location strings available in our sample. Merging these geocoding results over the location text to the data on profiles and tweets, we obtain the geographic locations for a total of 229 million tweets—26.4% of our analysis subset. This represents a 150-fold increase over the number of tweets geotagged with GPS coordinates by Twitter (see Section 3)^13^.

Perhaps surprisingly, the geocoded tweets were posted by only 6.23% (997,602 users) of all Twitter users in our dataset. A closer look at the data reveals the reason for this disproportion: Table 3 shows that Twitter users whose profile location could be matched with administrative regions in Germany were apparently much more active according to our data. However, the underlying reason for this discrepancy may not be the inactivity of users whose profile location could not be assigned to a region in Germany, which the data seem to suggest, but rather that this group may tweet less in German and therefore appear far less frequently in our dataset.

**Table 3:** Number of tweets per user from October 15, 2018, to October 14, 2021. Retweets and tweets from verified accounts are excluded.

	**Mean**	**Median**	**SD**	**Max**
Geocoded with profile location	230.0	9	1,939	792,298
Geotagged by Twitter	29.8	2	1,108	226,900
No geolocation	42.9	1	669	447,564

## 5. Evaluation

To evaluate the performance of our geocoding, we compare geocoding results with GPS geotags for the users for whom both these pieces of information are available, using common evaluation metrics (Section 5.1). Further, as studies have shown that the distribution of locations provided by Twitter via GPS tagging are biased in several dimensions (Malik et al., 2015; Arthur and Williams, 2019; Karami et al., 2021), we suspect similar issues with the geographic locations obtained via geocoding of user profile locations. To investigate this, we first look at whether geocoding via profile locations increases the potential bias in geolocated tweets by comparing the spatial distribution of users geolocated by Twitter and with our method (Section 5.2). Second, in Section 5.3, to assess whether geolocated tweets might differ in terms of content from non-geolocated tweets, we compare their respective bag-of-words distributions.

### 5.1. Geocoding Performance

Based on the assumption that GPS geotags from Twitter are the most reliable source of information about geographic locations, we use them as the basis for creating a gold standard to evaluate our geocoding results. Since GPS geotags are reported at the tweet level, the GPS–place-of-residence relation can be noisy. We apply several constraints when selecting the gold standard sample to ensure that locations provided by Twitter geotags and extracted from user profiles reflect the same underlying information (i.e., presumably the place of residence). Specifically, we select users for whom at least two geotags (which may refer to the same pair of coordinates) are covered by the same NUTS-3 region, and the geotags covered by said region account for more than half of all available geotags for the respective user. There are 13,423 users in our dataset whose geotags satisfy this condition and whose profile location could also be geocoded by our method^14^. The location to be used as the gold standard for a user is then calculated as the centroid of the geometry formed by all unique pairs of coordinates in the NUTS-3 region that covers the majority of that user's geotags.

We evaluate our geocoding results using four common metrics (Zheng et al., 2018): The first metric is accuracy, which treats location as discrete tokens and represents the percentage of cases in which the geocoded NUTS region matches the NUTS region containing the gold standard coordinates. The remaining three metrics are distance-based^15^, including accuracy@161, a relaxed accuracy metric that accepts results within a distance of 161 km (100 miles) from the gold standard as correct, as well as median and mean error distance of the geocoded regions to the gold standard.

Table 4 shows the evaluation results. Our geocoding procedure achieved over 90% accuracy at the NUTS-1 and NUTS-2 levels, and over 85 at the NUTS-3 level as well as when considering geocoding results at all levels combined. Over 95% of the geocoded NUTS regions are less than 161 km from the gold standard, with the median and mean error distances at 0 and 18.35 km, respectively^16^.

**Table 4:** Performance of our geocoding method.

				**Error distance (km)**	
**NUTS level**	**N**	**Accuracy**	**Accuracy@161**	**Median**	**Mean**
NUTS-1	13,423	92.74	-	-	-
NUTS-2	12,919	90.92	-	-	-
NUTS-3	12,793	86.07	-	-	-
All levels	13,423	85.70	95.87	0	18.35

### 5.2. Spatial Distribution of Geocoded Users

As suggested above, GPS coordinates are expected to show more variability at the user level. Our data support this assumption, as users with geotags provided by Twitter have more unique locations on average (mean: 2.54, standard deviation: 5.55) than users with locations geocoded by our method (mean: 1.04, standard deviation: 0.022)^17^. Nevertheless, since the median is 1 in both cases, we can assume that most users can be assigned to one NUTS-3 region, even in the case of the geographic locations provided by Twitter.

Following the general idea that most users can be assigned to one location, that is, their primary residence, we assign each user the statistical mode of their available locations—either geocoded with profile location or geotagged by Twitter. This allows us to unambiguously link Twitter user data to data from other sources (i.e., a user can only be attributed to one region when linking with official regional statistics). For example, if a user is assigned to Berlin ten times and to Munich three times (due to changes in their profile location over time), this user will be assigned to Berlin in our analysis. If a user has multiple modes of locations (i.e., multiple locations with the highest number of tweets associated with each of those locations), we draw a random location from those.

**Figure 3:**
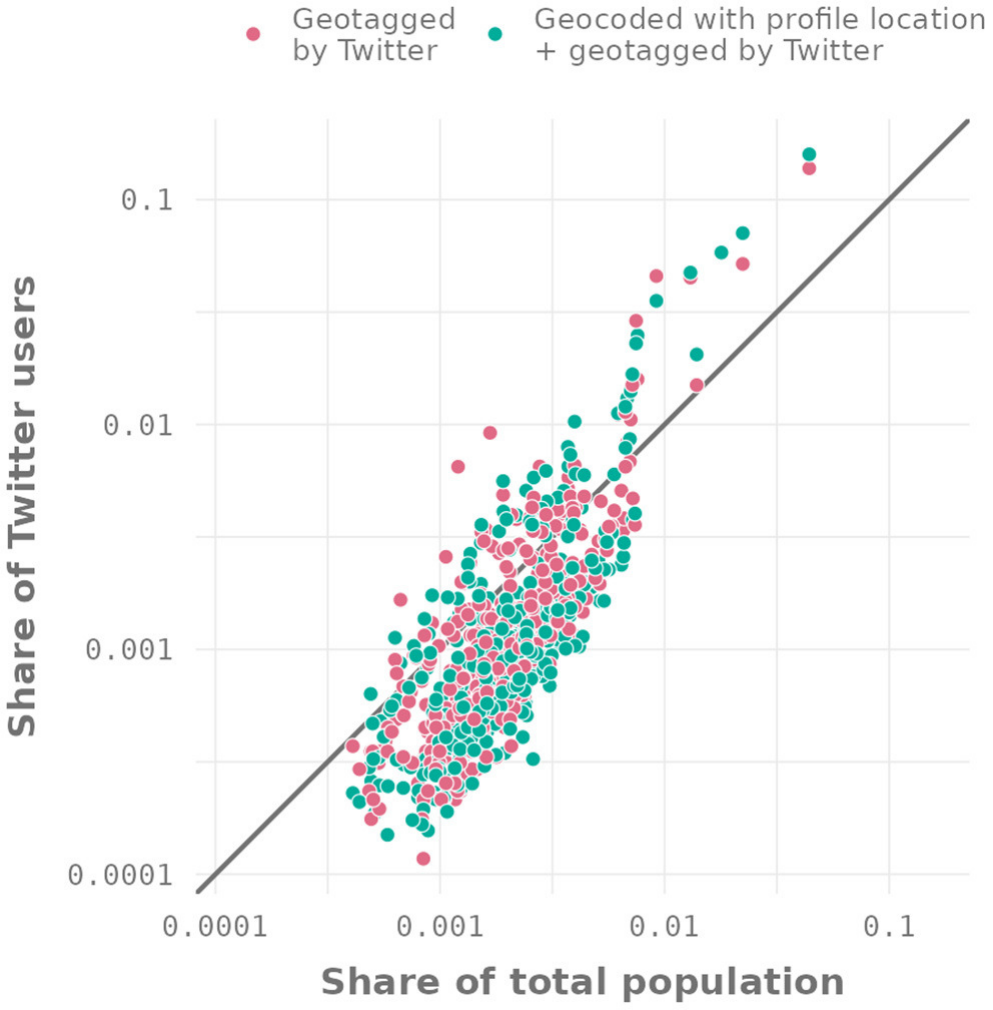
Share of Twitter users geotagged by Twitter and geocoded with profile locations vs. share of the German population by NUTS-3 region.

Figure 3 shows the distribution of locations provided by Twitter and by our method compared to the general population^18^. The share of Twitter users within a NUTS-3 region shows a rank similarity to the actual share of the real population in that region. However, after including newly geolocated users based on profile locations, we find the same biases as in the Twitter geotagged sample—that is, most smaller regions are slightly underrepresented, while a few larger regions (mostly cities) are overrepresented. On the other hand, the differences in percentage point between the two samples and the actual population are small. The average absolute error^19^—which corresponds to the average vertical distance of the points to the diagonal in Figure 3—is 0.00173 percentage points for Twitter geographic locations and 0.00111 for geographic locations obtained via the profile locations. This is possible evidence that the observable bias compared to the general population distribution is not from the GPS-based geographic locations, but instead represents a bias inherent to the platform, i.e., general self-selection into Twitter. Nevertheless, as our user sample is 20 times larger and our tweet sample is 150 times larger, it enables a wide variety of regional analyses at finer levels of granularity. Examples of regionalized content analyses can be found in the following sections.

### 5.3. Content of Non-geolocated and Geolocated Tweets

As previous research has shown, geolocated tweets may be susceptible to sampling bias (Malik et al., 2015), but it is not entirely clear whether this also applies to their content. To assess potential differences between the content of non-geolocated and geolocated tweets, we compare these two samples with two common metrics using a bag-of-words approach. A bag-of-words for a document, or in our case for a collection of tweets, contains the count of each word (“token”) after the data has been preprocessed and split into tokens.

We construct such a bag-of-words model, which we call “vocabulary,” in the form of a table containing the number of occurrences of each word in our data, using all tweets (whether geolocated or not). We decompose the tweets into individual tokens according to the following scheme: First, we use a regular expression to filter out all URLs in the data. Then, we employ a tokenizer that lowercases all words and excludes all characters that are not in the *letter, lowercase* subcategory of the Unicode 6.0 standard^20^—except for the octothorpe (#), since its use as a “hashtag” on Twitter signifies a special meaning if prefixing a token. During vocabulary building, words that occur fewer than 25 times in the whole dataset are excluded as they are mostly misspelled, made-up words or more or less randomly occurring strings. What remains is a vocabulary containing 2.2 million unique tokens.

For the comparison of non-geolocated and geolocated tweets, we create two sub-vocabularies containing the word counts for tweets without geolocation and the word counts for tweets geolocated either by our method or by Twitter. In creating these vocabularies, we restrict ourselves to the token pool of the full vocabulary and again remove words that occur less than 25 times in the full dataset. Sub-vocabularies may, however, contain words that occur fewer than 25 times if the word has a low frequency in our data and is spread across the two sub-vocabularies.

We compute two common metrics to compare our sub-vocabularies of non-geolocated and geolocated tweets: the Jaccard *S*_*J*_ coefficient and the cosine similarity *S*_*C*_. Since the Jaccard coefficient is the ratio between the size of the intersection of two sets and the size of their union, it measures the extent to which the sub-vocabularies contain the same words. It does not, however, take into account the distribution of words within the sets, that is, how many times a word occurs in each set. The cosine similarity is effectively calculated on the intersection of the two sets and is therefore agnostic to the set differences analyzed by the Jaccard coefficient, but can account for the word count differences within the intersection^21^. In our case, the Jaccard coefficient is *S*_*J*_(*Vocabulary*_*non*−*geo*_, *Vocabulary*_*geo*_) = 0.935, while the cosine similarity is *S*_*C*_(*Vocabulary*_*non*−*geo*_, *Vocabulary*_*geo*_) = 0.996. For both metrics, 1 represents the greatest possible similarity, and 0 the greatest possible dissimilarity. Although such summary statistics do not tell the whole story, they do show that the distribution of words in both data sets is extremely similar. The high Jaccard coefficient shows that both non-geolocated and geolocated tweets share more than 93% of words between them, with a large proportion of the words that are not shared across the vocabulary being odd words with rather low frequency (results not shown). The high cosine similarity supports this even more strongly. If the distribution of words among the common words were different in terms of their frequency, e.g., if some words were very prevalent in one corpus, but less common in the other (in relation to other words in the respective corpus), the cosine similarity would be low, which might ultimately indicate that some topics are less discussed or covered in one of the corpora. However, the very high cosine similarity is a strong indication that most words and (and possibly topics) are present to a similar extent in both non-geolocated and geolocated tweets.

## 6. Application Examples

In this section, we provide examples that demonstrate how regional variance observed in Twitter data can be used to approximate real-world behavior in the case of elections and regional party support, and how regional variance in dialects and gender-inclusive language can be captured in tweets. Furthermore, these simplified examples show that different types of analyses are possible at both the user and tweet level, and that digital behavior and communication correspond to known regional differences in the real world. In this respect, the forthcoming use cases display the potential of the geocoded data in sociological and political science analyses to reveal spatial variations in public discourse and behavior.

### 6.1. Voting Behavior and Party Support in Tweets

One advantage of our geocoding technique is that it significantly enhances the possibility for regionalized content analysis using Twitter data. Although analyses of regional differences in party support, political attitudes, and voting behavior have already been conducted with Twitter data (Beauchamp, 2017; Lopez et al., 2017), our data offer large gains in the number of cases available at the lower regional levels. Compared to survey data, analysis using Twitter data is comparatively inexpensive and can enable real-time tracking of regional public opinion (nowcasting)—a major challenge for survey projects (see Lopez et al., 2017).

To demonstrate the potential of this approach, we analyze hashtags in support for the German Green Party shortly before the September 2021 federal election and use party support on Twitter as a predictor of Green Party vote shares at the NUTS-2 level. For this purpose, we analyze data from the 30-day period (August 28, 2021, to September 26, 2021) leading up to the election on September 26, 2021, as this is the period when there is the most support and publicity for the party. First, we take data containing hashtags that indicated support for the Green Party^22^ and collect the count of users who tweeted using one of these hashtags at least once across the 38 NUTS-2 regions that we previously geocoded using the method presented above.

We compare the regional distribution of this quantity with the distribution of Green party votes in the 2021 federal election^23^. As we would expect a greater number of Twitter users who support the Green party as well as pro-Green votes in more populous regions, we divide both of our counts—the number of users tweeting in support for the Greens and the number of Green votes—by the total population at the NUTS-2 level. By doing this, both quantities are normalized by the same regional constant and, therefore, more comparable.

**Figure 4:**
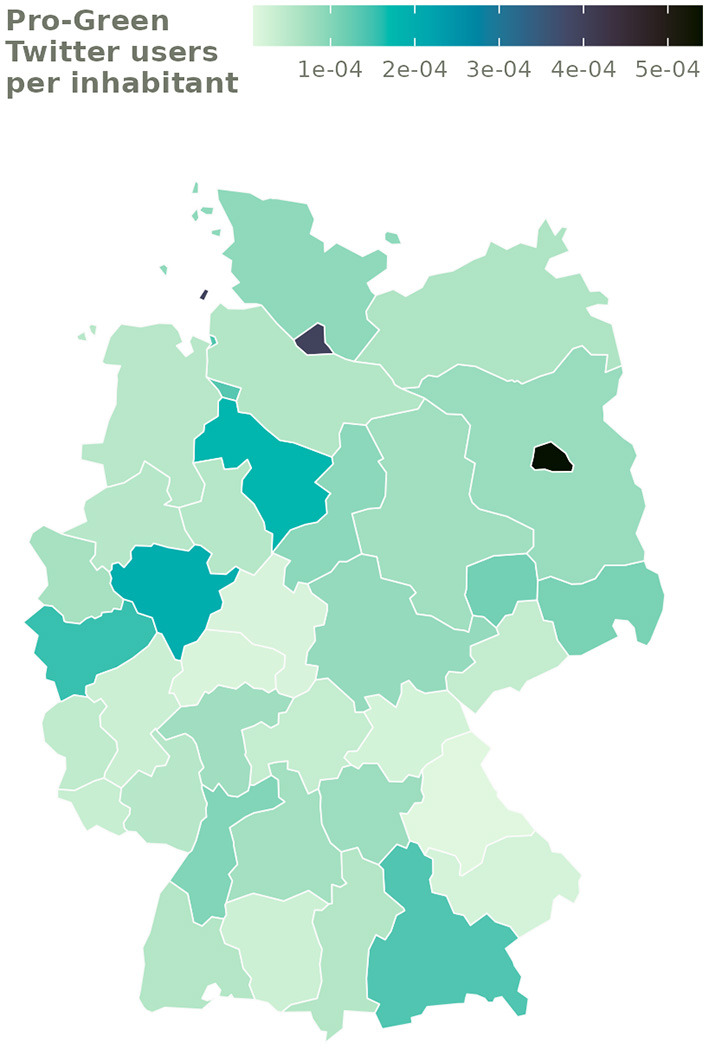
Number of users who tweeted in support for the Green party during the 30-day period leading up to the 2021 German federal election divided by population per NUTS-2 region.

The Pearson correlation coefficient for party support on Twitter and actual voting behavior at the NUTS-2 level shows a significant positive relationship between the two quantities [*r*_(35)_ = 0.528 at *p* < 0.001]. However, it is evident from Figure 4 that this correlation is in part driven by the two major cities of Berlin and Hamburg, which are overrepresented on Twitter and at the same time have comparatively strong levels of support for the Green party in the election. These results suggest that Twitter data geolocated by our method can—to some extent—provide an approximation for a known regional quantity, namely the level of electoral support for the Green Party in a given region in this example.

### 6.2. Regional Dialects

Like many other languages, German is characterized by different regional dialects. We perform a tweet-level analysis to capture linguistic differences in social media communication and investigate whether known regional dialects are represented in a similar pattern in digital communication.

An example of different dialects in Germany is the use of words for bread rolls, which are most commonly called *Brötchen*, but are usually called *Semmel* in southeastern Germany^24^. We test our data against this rather fuzzy concept of regional dialects, this time using data from the entire 3-year period covered by our dataset.

**Figure 5:**
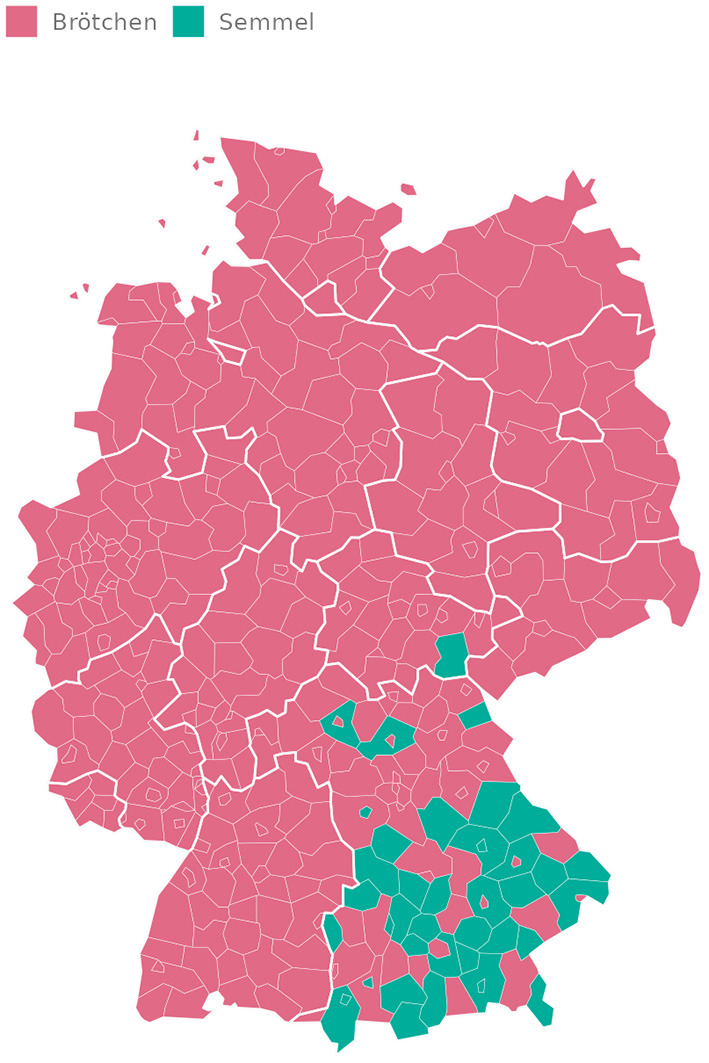
Most common name for German bread rolls by NUTS-3 region.

We search for tweets that mention bread rolls by performing a pattern match on a list of German names for bread rolls against our database (see Supplementary Section 3.2 for the list of patterns used). In this analysis, we do not normalize by the number of users and simply count the number of tweets that match one of the corresponding words describing a bread roll, as we are interested in the most frequently used expression by region. For each NUTS-3 region, we calculate the total number of occurrences of the above two terms for bread rolls in tweets that can be attributed to that region based on Twitter geotags or our geocoding results. Figure 5 shows the spatial distribution of the words *Brötchen* and *Semmel* across NUTS-3 regions. For each region, the word most frequently used in tweets by users from that region is shown. In 361 regions, *Brötchen* is the most frequently used word for bread rolls, while in 40 regions, *Semmel* is most often used. As expected, all regions that favor *Semmel* are located in southeastern Germany. Yet, even in a large part of southeastern Germany, *Brötchen* is still predominant, being a very common word that is widely known throughout Germany.

This example shows that, first, our data is able to capture regional variation in dialects, a concept rather difficult to quantify, especially when dealing with a word that is a common description known throughout Germany. Second, and more interestingly, in our example, regional variation cannot be captured as precisely if we aggregate tweets at the NUTS-2 level. In the NUTS-2 aggregate, *Brötchen* is more common than *Semmel* in all but two regions. This is due to the fact that even in southeastern Germany, there are many NUTS-3 regions where *Brötchen* is either more common, or less common but not significantly so. When aggregating at the NUTS-2 level, the total number of occurrences of *Brötchen* outweighs *Semmel*, despite the presence of subregions where *Semmel* is used more frequently. This exemplifies a case where finer-grained spatial analysis—enabled by the data geocoded with our method—allows for the uncovering of regional patterns that would otherwise go undetected.

### 6.3. Regional Variation in the Use of Gender-Inclusive Language

The German language uses gendered nouns, distinguishing three genders: masculine, feminine, and neuter. While there is an ongoing effort to make German more gender-neutral, both spoken and written German still tend to be biased toward masculine forms. Efforts to include all genders extend to the development of more gender-inclusive language. For example, the common noun *Mitarbeiter* (employees), a masculine plural noun, can be written in a more gender-inclusive way as MitarbeiterInnen, Mitarbeiter_innen, Mitarbeiter*innen, or *Mitarbeiter:innen*^25^. We show that our data can also be used to capture regional differences in the usage of gender-inclusive language. Here, we again aggregate users in our data who have used gender-inclusive plural nouns in at least one original tweet^26^, this time at the NUTS-3 level (401 regions). We divide this count by the number of unique users in each respective region to get an estimate of the share of users who use gender-inclusive language when tweeting.

**Figure 6:**
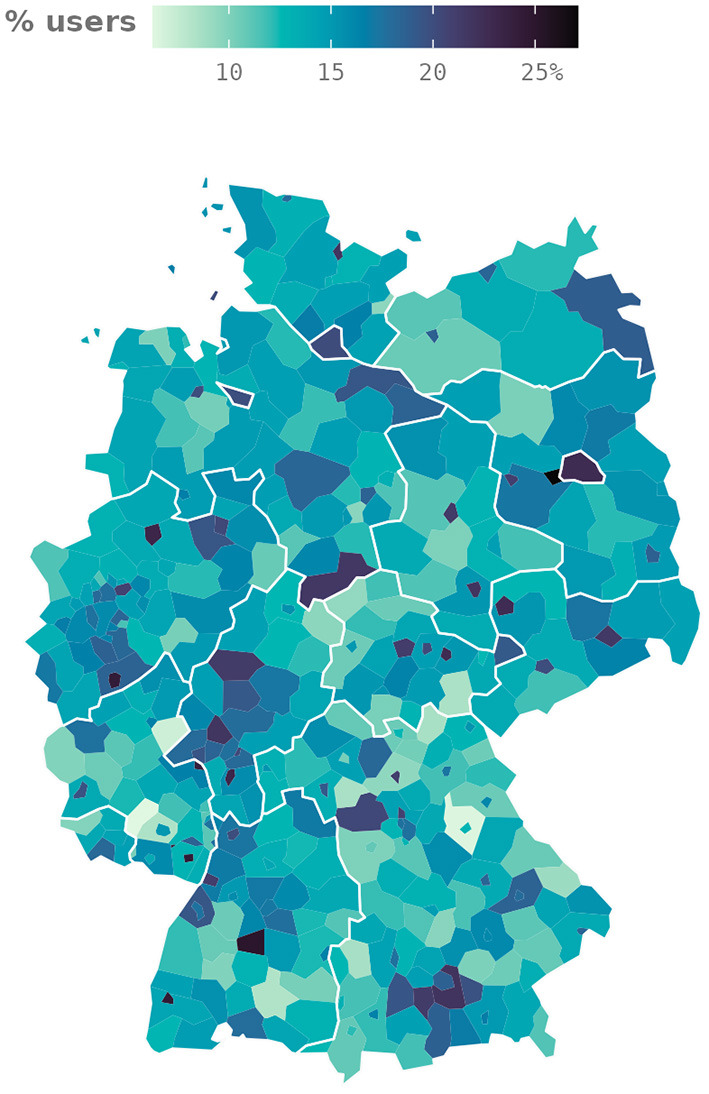
Percentage of Twitter users who used gender-inclusive language at least once by NUTS-3 region.

Figure 6 shows the distribution of the share of users who use gender-inclusive language across the 401 NUTS-3 regions. It is apparent that major cities tend to have higher shares of users tweeting with gender-inclusive forms of plural nouns. A possible hypothesis could be that Twitter users from cities are more gender-aware than users from rural areas. To assess this hypothesis, we calculate the Pearson correlation between the share of users using gender-inclusive language and the population density of the respective region. The resulting correlation coefficient *r*_(399)_ = 0.482 at *p* < 0.001 suggests that living in a less populous area may indeed be linked to less frequent use of gender-inclusive language.

A possible explanation for this correlation could be a larger share of academics or a larger young female population in urban areas. Combining data from INKAR (*Indikatoren und Karten zur Raum- und Stadtentwicklung*, English: indicators and maps of spatial and urban development) (Bundesinstitut für Bau-, Stadt- und Raumforschung, 2022) with our regional aggregates of Twitter data, we compute three linear regression models (Table 5) where the response variable in each case is the proportion of gender-inclusive language users in a region. Explanatory variables include the logarithm of population density (since the distribution of the population density is right-skewed), the proportion of employees with an academic degree, and the proportion of women aged 20–40 in the total population.

**Table 5:** Regression models of the proportion of gender-inclusive language users in NUTS-3 regions.

	**Model 1**	**Model 2**	**Model 3**	**Model 4**
(Intercept)	0.058^***^	0.082^***^	−0.110^*^	−0.106^*^
	(0.007)	(0.007)	(0.047)	(0.048)
Population density (log)	0.016^***^	0.003^*^	0.002	0.003
	(0.001)	(0.002)	(0.002)	(0.002)
Share academic employees		0.004^***^	0.004^***^	0.003^***^
		(0.000)	(0.000)	(0.000)
Share female population (20-40y)			0.004^***^	0.004^***^
			(0.001)	(0.001)
λ				0.185^**^
				(0.069)
R^2^	0.273	0.470	0.492	0.503
Num. obs.	401	401	401	401
Log likelihood	856.052	919.463	927.781	930.947

^***^p < 0.001; ^**^p < 0.01; ^*^p < 0.05.

Our results show a positive effect of population density on the share of gender-inclusive language users (Model 1). However, the inclusion of the share of employees with an academic degree (Model 2) leads to a positive and significant effect of this predictor as well as a substantial increase in explanatory power, while the effect of population density diminishes. Finally, when the proportion of women aged 20–40 is added as a covariate (Model 3), which also has a significant positive effect, the effect of population density becomes no longer significant. This suggests that the correlation between population density and gender-inclusive language is indeed an effect of the demographic structure of the NUTS-3 regions.

Examining the residuals of the OLS models reveals the presence of spatial autocorrelation, with Moran's *I* significant at *p* < 0.05 in all three models. This suggests potential biases in the estimation of parameters in the presented linear models. To account for spatial dependence in the unobservables, we add a spatial autoregressive error term (Model 4)^27^. While the λ parameter is positive and significant, indicating spatial clustering among the unobserved characteristics, the coefficients of the spatial error model for the independent variables remain very similar to those of the OLS model, further supporting the results reported in the previous paragraph on the effects of the proportion of academics and young female population on the use of gender-inclusive language.

## 7. Discussion

Digital behavioral data and big data are becoming an increasingly important resource for social science research. In this respect, Twitter is one of the most widely used data sources, not least because of the ease of access to the data for research purposes.

In this paper, we implemented a method for geocoding Twitter users and tweets using the user profile locations to substantially increase the amount of Twitter data usable for regional analyses. By using a self-hosted, customized database of the OpenStreetMap search engine Nominatim to geocode profile locations in our dataset of German tweets, we achieved an 150-fold increase in the number of tweets that can be geolocated in Germany, from 0.18 to 26.4%. With the new, larger sample, we were able to confirm the biases in the spatial distribution of Twitter users highlighted in previous research, with larger cities overrepresented, and smaller cities and rural areas underrepresented compared to the actual population. We developed and maintain a companion free open-source R package, nutscoder (github.com/long39ng/nutscoder), which facilitates straightforward reuse of our geocoding procedure and extends the applicability of our method to administrative regions outside Germany.

We evaluated our geocoding results based on a number of parameters. First, the assessment of the geocoding performance based on comparisons of geocoded profile locations and geotags provided by Twitter showed a high level of accuracy of our results. Second, the geolocated and non-geolocated tweets do not appear to differ systematically in terms of word occurrences. Consequently, tweets geolocated using our method could represent an almost random subsample of all tweets for many applications. However, further analysis is needed to assess the potential bias in the content of geolocated tweets compared to non-geolocated tweets.

Moreover, we have demonstrated through a number of use cases that our geolocated data are able to capture a) known regional differences (predicting party votes on the regional level), b) fuzzy regional differences (reproducing the spatial distribution of known regional dialects), and c) previously unknown regional differences, for example in the use of gender-inclusive language between urban and rural areas.

Many other applications of analyzing regionalized Twitter data are potentially possible, including monitoring regional changes in attitudes and behavior over time, deriving proxy information about regions that can be used as explanatory variables. In particular, when research aims to compare small regions or small time periods, survey data are usually not suitable, and indicators derived from Twitter data may be able to fill certain data gaps. Thus, although Twitter does not allow for deriving population parameter estimates in almost all cases, it can be useful for a number of research applications and should be further studied and evaluated by social science methodology research.

By standardizing the geocoding results to official codes of administrative regions, our procedure makes it simple to combine the geocoded data with regional data from other sources, such as official statistics. This approach also has the additional benefit of being less privacy-sensitive compared to exact point coding. Of course, the geocoding output is not limited to administrative regions. By customizing the target geographic data on which we perform spatial joins of the geocoding results, we can modify the output to any desired set of regional identifiers.

Compared to approaches that model Twitter user networks and tweet content to infer users' real-world locations, our method of geocoding the profile location text should be able to provide more reliable results at much higher speed. Since we only geocode the information that explicitly relates to the users' locations, our geocoding results have a much lower degree of uncertainty and require much less effort to validate compared to the above alternatives. This makes our geocoding method particularly suitable for applications that work with very large amounts of data and/or in real time. Moreover, using our method to obtain more geographic information based on user profile locations provides more data that can be used for both training and evaluation of more sophisticated methods, thereby improving the efficacy of these methods. Given that many users do not provide profile locations— and many of those who do, do not provide actual locations—more sophisticated, specialized geolocation methods are the likely next step that will allow us to achieve better spatial coverage of Twitter data in future studies.

## Data Availability Statement

The data analyzed in this study is subject to the following licenses/restrictions: Redistribution of the collected Twitter data is restricted by the Twitter Terms of Service, Privacy Policy, Developer Agreement, and Developer Policy. The IDs of the geocoded tweets and the geocoding results associated with those IDs are available at gitlab.ub.uni-bielefeld.de/geocoding-german-twitter/geocoded-tweets. The geocoding procedure can be reproduced with the code in the paper's GitLab repository (gitlab.ub.uni-bielefeld.de/geocoding-german-twitter/geocoding-german-twitter) and/or with the use of the companion R package nutscoder (github.com/long39ng/nutscoder). Requests to access these datasets should be directed to HLN, long.nguyen@uni-bielefeld.de.

## Ethics Statement

Ethical review and approval was not required for the current study in accordance with the local legislation and institutional requirements. Written informed consent for participation was not required for this study in accordance with the national legislation and the institutional requirements.

## Author Contributions

SKü, HLN, and DT conducted the literature review. HLN designed and implemented the geocoding. HLN and DT performed the evaluation of the geocoding results. DT and AK performed the analyses in the application examples. SKn managed the infrastructure for data collection, management, and analysis. All authors discussed the results and contributed to the final version of the manuscript.

## Funding

This research was partly funded by (a) the Leibniz ScienceCampus SOEP RegioHub (Bielefeld University and SOEP/DIW Berlin) and (b) the German Ministry for Family Affairs, Senior Citizens, Women and Youth (BMFSFJ) in the context of the National Discrimination and Racism Monitor (NaDiRa) as well as the Research Association Discrimination and Racism (FoDiRa) of the DeZIM Research Community (German Center for Integration and Migration Research).

## Conflict of Interest

The authors declare that the research was conducted in the absence of any commercial or financial relationships that could be construed as a potential conflict of interest.

## Publisher's Note

All claims expressed in this article are solely those of the authors and do not necessarily represent those of their affiliated organizations, or those of the publisher, the editors and the reviewers. Any product that may be evaluated in this article, or claim that may be made by its manufacturer, is not guaranteed or endorsed by the publisher.

## Footnotes

^1^To be precise, GPS is one specific satellite system for obtaining user locations. Modern technology also makes use of other systems such as GLONASS, Galileo, QZSS and BeiDou for this purpose. In this paper, however, we use GPS as an umbrella term to refer to this kind of technology.

^2^NUTS (*Nomenclature des unités territoriales statistiques*) is “a common classification of territorial units to enable the collection, compilation and dissemination of harmonized regional statistics in the EU and the UK.” The NUTS system has a hierarchy of three levels. In Germany, NUTS-1 is federal states (*Bundesländer*), NUTS-2 is government regions (*Regierungsbezirke*), and NUTS-3 is districts (*Kreise*) or major, district-free cities (*kreisfreie Städte*) (European Commission, 2016).

^3^This percentage refers to the number of users who sent English tweets collected in Alex et al. (2016). Analysis of our own dataset (Section 3.2) shows a similar proportion of Twitter users who provide a profile location.

^4^ www.geonames.org

^5^ developer.twitter.com/en/docs/twitter-api

^6^Our list of the most common German words was compiled from the word list DeReKo-2014-II-MainArchive-STT.100000 (www.ids-mannheim.de/digspra/kl/projekte/methoden/derewo/), from the Institute for German Language (www.ids-mannheim.de). Note that Twitter does not allow queries that filter tweets based solely on Twitter's language recognition. Therefore, it is necessary to provide a list of additional keywords or parameters—in our case, the 100 most common German words. A list of these words can be found in the (Supplementary Section 1).

^7^Retweeting is the act of sharing another user's tweet publicly on Twitter.

^8^These profile location strings are obfuscated to protect the privacy of users.

^9^This definition is more conservative than the more common definition of active users, which also counts registered users who visited the platform but did not post anything.

^10^Due to the first and last date of the analysis subset being in the middle of the month, the first and last month has a substantially lower number of active users.

^11^The Nominatim database instances are containerized with Docker (image from github.com/mediagis/nominatim-docker) and deployed via Kubernetes, each with a maximum of 64 CPU threads and 16 GB of shared memory. Wikipedia data is imported into both instances to leverage the result ranking mechanism described above. PostGIS (postgis.net) is enabled in both Nominatim databases as well as the Twitter database to facilitate geocomputational operations.

^12^A more obvious setup would be to simply geocode all user profile locations in one pass with the global Nominatim instance. However, by first running all profile location strings through the German Nominatim instance, we can filter out a large number of irrelevant strings (i.e., non-locations or locations not in Germany) in much less time, since the German database is significantly smaller than the worldwide database (100 GB vs. 1.4 TB). Obviously, after this step, the profile location strings—now substantially fewer—still have to be geocoded with the global instance of Nominatim, since the Germany-only instance—due to the lack of data on places outside Germany—mistakes place names like “New York” for locations in Germany.

^13^The numbers presented in this section refer to the results of geocoding Twitter profile locations using administrative regions in Germany. This means that valid profile locations, that is, those that contain actual place names, but do not refer to locations in Germany, do not yield any results. In applications where locations outside Germany are also taken into account, the coverage provided by the geocoded user profile locations is likely to be much higher.

^14^An evaluation based on all users for whom Twitter geotags are available and whose profile locations could be geocoded by us (i.e., without the restrictions to filter for users in the gold standard sample in this section) is reported in the Supplementary Section 2.

^15^For these metrics, we calculate the distance between the gold standard point and the polygon of the geocoded NUTS region for each case.

^16^While these numbers appear to show much better overall performance compared to other methods of geocoding using Twitter profile locations, such as in Dredze et al. (2013), meaningful comparison is not possible, since they performed the geocoding on a much smaller sample of tweets that were posted by users from another country.

^17^We count the number of locations per user at the NUTS-3 level. This means that for a user, unique pairs of geocoordinates that fall within a NUTS-3 region are counted as a single location.

^18^Source: Destatis (2021).

^19^The absolute error for a NUTS-3 region is calculated as the absolute difference between the region's actual share of population and the share of Twitter users in the region. For example, if a NUTS-3 region has 0.01% of the actual population, but only 0.009% of Twitter users, the absolute error for this region is |0.01−0.009| = 0.001.

^20^The Unicode 6.0 standard includes 1,759 lowercase letters from multiple languages in its specification (www.unicode.org/versions/Unicode6.0.0/UnicodeStandard-6.0.pdf).

^21^The cosine similarity, interpreted for the case at hand, corresponds to the angle between the vocabularies, that is, the vectors of term frequencies. Hence, despite the difference in size in the absolute values between the partial vocabularies, no further normalization is necessary.

^22^The hashtags we use are: #diesmalgrün (#thistimegreen), #grünwählen (#electgreen), #bereitweilihresseid, (#readybecauseyouare), #grün (#green), #grüne (#greens) and the respective version with mutated vowels replaced (The full query to the database can be found in the Supplementary Section 3.1). It should also be noted that hashtags like #green or #greens are often used in news reports and may not represent actual support for the party. However, because we excluded retweets and verified Twitter accounts, which are mostly a superset of professional accounts such as news outlets, we assume that these hashtags much more accurately represent party support.

^23^We use the second vote (*Zweitstimme*), which voters cast for a party at the national level, not for a regional candidate. Since party votes are only available at the district level (Der Bundeswahlleiter, 2021), we aggregate these election results at the NUTS-2 level. We also exclude Saarland, where it was not possible to cast a second vote for the Greens in the 2021 federal election (tagesschau.de, 2021).

^24^Other variations also exist across Germany, but they occur much less frequently compared to these two.

^25^This list of possible variants is exhaustive.

^26^The regex pattern to query usage of gender-inclusive language is reported in the Supplementary Section 3.3.

^27^Spatial error model: **y** = **X**β+**u**, where **u** = λ**Wu**+ε and **W** is the spatial weights matrix (Rüttenauer, 2022).
