# Corrigendum: Efficient and reliable geocoding of German Twitter data to enable spatial data linkage to official statistics and other data sources

**DOI:** 10.3389/fsoc.2022.995770

**Published:** 2022-08-02

**Authors:** H. Long Nguyen, Dorian Tsolak, Anna Karmann, Stefan Knauff, Simon Kühne

**Affiliations:** Faculty of Sociology, Bielefeld University, Bielefeld, Germany

**Keywords:** Twitter, geocoding, spatial linkage, official statistics, regional analysis

In the original article, there was an error in the Funding statement. The funder “Leibniz ScienceCampus SOEP RegioHub (Bielefeld University and SOEP/DIW Berlin)” was missing. The correct Funding statement appears below.

## Funding

This research was partly funded by (a) the Leibniz ScienceCampus SOEP RegioHub (Bielefeld University and SOEP/DIW Berlin) and (b) the German Ministry for Family Affairs, Senior Citizens, Women and Youth (BMFSFJ) in the context of the National Discrimination and Racism Monitor (NaDiRa) as well as the Research Association Discrimination and Racism (FoDiRa) of the DeZIM Research Community (German Center for Integration and Migration Research).

The authors apologize for this error and state that this does not change the scientific conclusions of the article in any way. The original article has been updated.

## Publisher's note

All claims expressed in this article are solely those of the authors and do not necessarily represent those of their affiliated organizations, or those of the publisher, the editors and the reviewers. Any product that may be evaluated in this article, or claim that may be made by its manufacturer, is not guaranteed or endorsed by the publisher.

