# Supplementary material for: Efficient and Reliable Geocoding of German Twitter Data to Enable Spatial Data Linkage to Official Statistics and Other Data Sources
Source: Front Sociol. 2022 Jun 9;7:910111. doi: 10.3389/fsoc.2022.910111 (PMC9220088; doi:10.3389/fsoc.2022.910111)
Supplement: Supplementary file 1 [file Data_Sheet_1.PDF]

Supplementary Material

1 100 MOST COMMON GERMAN WORDS USED TO QUERY THE TWITTER API

der	zu	einer	Das	habe
die	es	einem	durch	sei
und	als	einen	wir	wenn
in	er	über	das	Jahr
den	am	wurde	der	zwei
von	bei	werden	kann	Jahren
mit	die	so	gegen	seine
ist	Der	nur	hatte	sein
im	an	oder	unter	gibt
für	hat	um	vom	ihre
des	sie	zur	bis	Er
sich	aus	vor	Es	immer
nicht	sind	ich	mehr	beim
Die	wird	man	schon	Ein
auf	nach	haben	wieder	Und
dem	dass	In	werden	dieser
das	war	Sie	Ich	wie
ein	zum	wie	Das	wurden
eine	zu	Uhr	Im	keine
auch	noch	aber	dann	soll

2 GEOCODING PERFORMANCE WHEN COMPARED TO ALL USERS WITH TWITTER GEOTAGS

(That is, without applying the constraints for the gold standard described in Section 5.1.)

				Error Distance (km)	
NUTS level	N	Accuracy	Accuracy@161	Median	Mean
NUTS-1	26959	84.73	-	-	-
NUTS-2	25635	81.43	-	-	-
NUTS-3	25305	73.60	-	-	-
All levels	26959	72.56	90.39	0	40.73

Table S1. Evaluation metrics measured based on all available geotags.

3 REGEX PATTERNS FOR TWEET SEARCHES

3.1 Green party support

```
flags = gi
((?:(^|\W))#grun(?:($|\W)))|
((?:(^|\W))#gruen(?:($|\W)))|
```

```
((?:(^|\W))#grüne(?:($|\W)))|
((?:^|\W)#gruene(?:$|\W))|
((?:^|\W)#diesmalgrün(?:$|\W))|
((?:^|\W)#diesmalgruen(?:$|\W))|
((?:^|\W)#grünwählen(?:$|\W))|
((?:^|\W)#gruenwaehlen(?:$|\W))|
((?:^|\W)#bereitweilihresseid(?:$|\W))
```

3.2 Regional dialects

```
flags: -gi
((?:(^|\W))brötchen(?:($|\W)))|
((?:(^|\W))broetchen(?:($|\W)))|
((?:(^|\W))semmel(?:($|\W)))|
((?:(^|\W))semmerl(?:($|\W)))|
((?:(^|\W))semmeln(?:($|\W)))|
((?:(^|\W))semmerln(?:($|\W)))|
((?:(^|\W))weck(?:($|\W)))|
((?:(^|\W))wecke(?:($|\W)))|
((?:(^|\W))weckle(?:($|\W)))|
((?:(^|\W))weckerl(?:($|\W)))|
((?:(^|\W))weckerle(?:($|\W)))|
((?:(^|\W))schrippe(?:($|\W)))|
((?:(^|\W))kipf(?:($|\W)))
```

3.3 Gender-inclusive speech

```
flags: none
((?:(^|[a-zA-Z]))In(?:($|\W)))|
((?:(^|[a-zA-Z]))Innen(?:($|\W)))|
((?:(^|[a-zA-Z]))_in(?:($|\W)))|
((?:(^|[a-zA-Z]))_innen(?:($|\W)))|
((?:(^|[a-zA-Z])):in(?:($|\W)))|
((?:(^|[a-zA-Z])):innen(?:($|\W)))|
((?:(^|[a-zA-Z]))\*in(?:($|\W)))|
((?:(^|[a-zA-Z]))\*innen(?:($|\W)))
```

4 MAP OF REGIONAL DIALECTS FOR “BREAD ROLLS”

This is an alternative map to Figure 5 and contains more German terms for “bread rolls”. The underlying data for this figure are the tweets queried by the regex pattern specified in Section 3.2 of the Supplementary Material.

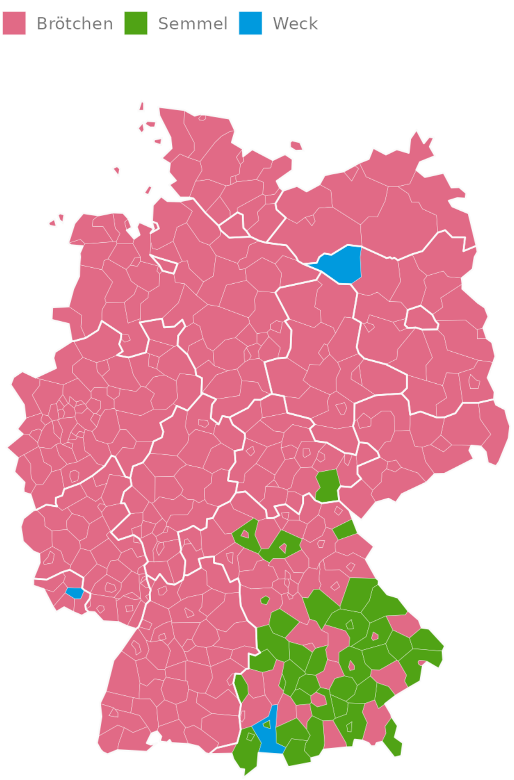

Figure S1. Most common name for German bread rolls by NUTS-3 region
